# Inflammatory Cytokines in Psoriatic Arthritis: Understanding Pathogenesis and Implications for Treatment

**DOI:** 10.3390/ijms241411662

**Published:** 2023-07-19

**Authors:** Bong-Woo Lee, Su-Jin Moon

**Affiliations:** 1 Division of Rheumatology, Department of Internal Medicine, Seoul St. Mary’s Hospital, College of Medicine, The Catholic University of Korea, Seoul 06591, Republic of Korea; tdomu1029@gmail.com; 2 Division of Rheumatology, Department of Internal Medicine, Yeouido St. Mary’s Hospital, College of Medicine, The Catholic University of Korea, Seoul 07345, Republic of Korea

**Keywords:** psoriatic arthritis, inflammatory cytokines, tumor necrosis factor-alpha, interleukin-17, interleukin-23, JAK/STAT signaling pathway

## Abstract

Psoriatic arthritis (PsA) is a persistent, inflammatory disease that affects individuals with psoriasis, arthritis, and enthesitis. Research has demonstrated that inflammatory cytokines such as tumor necrosis factor-alpha (TNF-α), interleukin-23 (IL-23), and interleukin-17 (IL-17) play a pivotal role in both the onset and progression of PsA. These cytokines are generated by activated immune cells and stimulate the attraction of inflammatory cells to the synovium and joint tissues, resulting in the deterioration of cartilage and bone. The blocking of these cytokines has become a successful treatment strategy for PsA, as biological drugs that inhibit TNF-α, IL-23, and IL-17 have demonstrated notable clinical benefits. The association between PsA and other types of inflammatory cytokines or chemokines, excluding TNF-α, IL-23, and IL-17, has been extensively investigated in numerous studies. These findings may provide a chance for the discovery of novel therapeutic agents targeting other molecules, distinct from the currently approved biologics and targeted synthetic disease-modifying anti-rheumatic drugs. In this review, we discuss the current understanding of the role of inflammatory cytokines in PsA pathogenesis and clinical implications of targeting these cytokines for PsA treatment.

## 1. Introduction

Psoriatic arthritis (PsA) is a persistent condition associated with immune-related inflammation that affects joints and other elements of the musculoskeletal system, in addition to skin manifestations. It is estimated that this disease affects approximately 1–2% of the worldwide population [1]. PsA is recognized as part of psoriatic diseases and belongs to a complex group of spondyloarthritides (SpA) [2,3,4].

PsA is identified by a varied range of clinical manifestations, such as peripheral joint inflammation, inflammatory back pain, uveitis, enthesitis, tenosynovitis, psoriasis, and nail changes [5]. Historically, PsA was regarded as a relatively benign condition. However, it is now widely acknowledged that the functional impact of PsA is comparable to that of other types of inflammatory arthritis, including rheumatoid arthritis (RA) and axial SpA (axSpA) [1]. In many individuals with PsA, the disease exhibits characteristics of progressive and destructive changes. Significant pathological changes occur already in the early stages of PsA, with approximately half of the patients displaying structural damage within 2 years from disease onset [6]. Research has demonstrated that a delay in diagnosing PsA is linked to unfavorable outcomes impacting the quality of life [7].

While the precise cause of PsA is not fully comprehended, it is believed to arise from a complex interplay of environmental, genetic, and immunological factors [2]. Specific human leukocyte antigen (HLA) alleles, namely HLA-B*27, HLA-B*08, HLA-B*38, and HLA-B*39, exhibit a strong correlation with the prevalence of PsA [8]. It is hypothesized that these alleles may render individuals more susceptible to PsA by modifying the immune response to self-antigens. Furthermore, environmental factors, including infections and mechanical stress, can potentially act as triggers for PsA development in individuals with a genetic predisposition [9,10]. The microbiome of the gut and skin might have an impact on the development of PsA [11,12]. Immunological factors, including cytokines and chemokines, are believed to have a crucial role in PsA pathogenesis. Tumor necrosis factor-alpha (TNF-α), interleukin-17 (IL-17), and interleukin-23 (IL-23) have been recognized as significant contributors to the inflammatory response, tissue damage, and bone erosion associated with PsA. Therapies targeting these cytokines have been developed and used as treatments for patients with PsA. Despite advancements in comprehending the molecular mechanisms that cause PsA, the existing knowledge is not sufficient to meet significant clinical needs in effective treatments [13]. Overall, PsA pathogenesis is complex and multifactorial, involving genetic, environmental, and immunological factors. 

The improved understanding of PsA pathogenesis has resulted in the development of powerful biologics and small-molecule drugs that selectively target cytokines and signaling pathways. However, due to the complex pathogenesis of PsA involving both innate and acquired immune responses, as well as the highly heterogeneous presentation of the disease among individuals, many current treatments fall short of achieving the desired outcome. For this reason, further research on the roles of other inflammatory cytokines in the pathogenesis of PsA could contribute to the search for valid diagnostic biomarkers, factors for predicting treatment response, and new target molecules for treating this disease. In this review, we summarize the latest information about inflammatory cytokines and possible therapeutic target molecules in PsA. 

## 2. Immune Cells in PsA Pathogenesis

PsA is a chronic and complex condition characterized by the involvement of the skin, enthesis, and synovium. The degree of this involvement may vary among individuals and is influenced by genetic and environmental factors [14]. PsA is initiated by the stimulation of dendritic cells (DCs) and macrophages. These cells expose antigens to T cells through the toll-like receptor-2 (TLR-2) signaling pathway, facilitated by major histocompatibility complex (MHC) I. This activation occurs in response to a combination of genetic predisposition, environmental conditions, and biophysical factors. Consequently, the activation of both innate and adaptive immune responses leads to the increased secretion of various cytokines, such as IL-1, IL-6, TNF-α, IL-17, and IL-23 [15]. In addition, the activation and invasion of T cells and macrophages are considered essential in the initiation of inflammatory and destructive processes in the joints [16].

Specifically, DCs induce T cell differentiation by presenting antigens and secreting various pro-inflammatory cytokines. Increased expression of TLR-2 has been detected in the immature DCs of PsA patients [17]. Patients with PsA exhibit an elevated proportion of immature myeloid DCs compared to plasmacytoid DCs in their synovial fluid [18]. In PsA, TLR stimulation leads to the polarization of T cells towards the Th1 subset, subsequently increasing the production of TNFα, IFN-γ, and IL-2 [17]. Plasmacytoid DCs generate cytokines, including IFN-γ, TNF-α, IL-12, and IL-23, which subsequently serve as signals for the clonal proliferation of CD4^+^ and CD8^+^ T cells [19].

Activated macrophages participate in various pro-inflammatory processes within the synovium. A reduction in the quantity of CD68^+^ macrophages was observed in the synovium of patients with PsA who responded to treatment, which highlighted the role of these cells in PsA pathogenesis [20]. Skin inflammation induced by a TLR-7 ligand in murine models was associated with the infiltration of CD68^+^ macrophages and stimulation of inflammatory cytokines expression in the joints. This study implied that macrophages could contribute to the inflammatory process in PsA and may be a crucial factor in the skin-to-joint inflammation transition [21]. In RA, M1 macrophages become prevalent, which leads to the secretion of high levels of pro-inflammatory cytokines, activation of T and B cells through antigen presentation, and stimulation of bone resorption. Similarly, the administration of a TLR-7 agonist increases the M1/M2 macrophage ratio in psoriatic skin lesions [22]. On the contrary, there is no difference in M2 cytokine expression between PsA and RA, but PsA is marked by lower expression levels of M1 cytokines compared to those in RA [23]. 

Mast cells located in the synovium induce angiogenesis, neutrophil recruitment, and proliferation of synovial fibroblasts, indicating that these cells may actively contribute to the inflammatory arthritis [24]. In the synovium of peripheral SpA, mast cells are the predominant source of IL-17A, whereas only a small number of IL-17-positive T cells was identified [25]. Likewise, tissue-resident mast cells may increase inflammation by producing IL-17A. However, it remains to be established in more detail how the release of IL-17A from mast cells is controlled in the affected tissue during inflammation [26].

Other various cells in the synovial tissue could potentially participate in PsA pathogenesis. Notably, elevated levels of type 3 innate lymphoid cells (ILCs), which produce IL-17 and IL-22, have been observed in the synovial fluid of PsA patients [27]. Conversely, there was a decrease in the number of type 2 ILCs, which typically generate IL-4, IL-5, IL-9, and IL-13. Significantly, the ratio of type 2 ILCs to type 3 ILCs showed a strong correlation with both clinical indicators of disease activity and the radiographic evidence of arthritis and bony destruction. In addition, it has been shown that environmental stress signals activate natural killer (NK) cells, leading to their involvement in the harmful processes associated with PsA development [28,29]. Additionally, IL-15 effectively stimulates dormant NK cells within the synovium, causing them to transition into an effector phase [30].

In the development of PsA, innate immune cells contribute by producing IL-12 and IL-23, which stimulate the differentiation of T cells into distinct subtypes referred to as Th1 and Th17 helper T cells [5,31]. Consequently, IL-22 and IL-17 are secreted, which in turn increase the secretion of TNF-α [32]. T cells are the primary contributors to PsA pathogenesis, as evidenced by the elevation of CD4^+^ T17 cells that secrete IL-23 and IL-17 in both the peripheral blood and synovial fluid [33,34,35]. Furthermore, an accumulation of cells producing IL-17 was detected within the affected joints of PsA patients, and this group may also include IL-17 secreting CD8^+^ T cells [36]. Another study conducted on PsA patients unveiled a notable increase in the quantity of memory CD8^+^ T cells within the synovial fluid, surpassing their levels detected in the peripheral blood. Additionally, these cells were identified as active CD8^+^ T cells expressing the CXCR3 receptor. Moreover, elevated levels of CXCR3 receptor ligands, such as CXCL9 and CXCL10, were also observed in the synovial fluid [37]. Th1, Th17, and CD8^+^ cytotoxic T cells have been recognized as crucial contributors to the inflammatory processes in PsA, suggesting the existence of distinct immune cellular pathways in this disease [13]. Generally, regulatory T cells (Tregs) are thought to participate in suppressing chronic inflammation and promoting an anti-inflammatory response. However, research on psoriasis and PsA has yielded conflicting findings regarding the relationship between Treg levels and disease activity [38,39,40]. The specific function of B cells is not fully understood; however, they can present antigens to T cells [5].

In summary, interactions between diverse types of immune cells lead to the production of various cytokines, such as IL-23, TNF-α, IL-17 (predominantly IL-17A isoform), and IL-22, which promote inflammation and stimulate tissue-resident cells in the joint and enthesis. These cells, including fibroblast-like synoviocytes, chondrocytes, osteoblasts, and osteoclasts, cause the degradation of cartilage, bone erosion, and joint destruction by secreting extracellular matrix degradation enzymes and the receptor activator of nuclear factors’ κB ligand (RANKL). Furthermore, upon stimulation, these cells secrete pro-inflammatory mediators to attract additional immune cells, thereby establishing a sustained immune response in patients with active PsA [5].

## 3. Key Inflammatory Cytokines in PsA 

In PsA, inflammation is triggered by cytokines released by activated immune cells (Table 1). Over the past few years, there has been a notable change in research focus regarding the key factors that contribute to the development of PsA. Previously, the focus was primarily on TNF-α, and cytokines associated with the Th1 immune response. However, more recent studies have shifted their attention to Th17 cells, as well as IL-23 and IL-17 cytokines. The administration of monoclonal antibodies targeting specific cytokines, such as IL-23, IL-17A, and TNF-α, has been proven to effectively attenuate inflammation and ameliorate clinical manifestations of PsA. It is highly probable that each distinct cytokine possesses a unique pathophysiological role in the development and progression of the disease. IL-23 appears to play a role in initiating the development of the disease, whereas IL-17A primarily enhances the disease process. On the other hand, TNF-α plays a critical role in carrying out downstream effects in the pathogenesis of PsA [41].

### 3.1. Role of TNF-α in PsA

TNF-α, a pro-inflammatory cytokine produced by cells like macrophages and monocytes, is prominently present during inflammatory processes [42]. It serves as a key component of pro-inflammatory immune responses. TNF-α interacts with two distinct receptors, TNFR1 and TNFR2, initiating separate signaling pathways that trigger apoptosis, cell differentiation, proliferation, and migration. As a result, TNF-α contributes to the development of inflammatory reactions. Additionally, TNF-α plays a crucial role in promoting bone resorption by directly activating osteoclast precursor cells via the RANKL signaling pathway [43]. At high concentrations, TNF-α causes adverse effects, including uncontrolled inflammatory responses, increased formation of osteoclast precursors, and enhanced osteoclast activity, ultimately resulting in bone resorption [44].

TNF-α plays a pivotal role in the pathogenesis of SpA manifestations, encompassing skin, enthesis, joint, and spine involvement, which can be considered as the secondary effects of hyperactivated TNFα-dependent inflammation due to the activation of both innate and adaptive immune responses [45]. The evidence supporting the involvement of TNF-α in inflammatory forms of arthritis is particularly compelling [43]. Elevated levels of TNF-α are detected in the synovial fluid and tissues of patients with RA and PsA. Various cell types, including macrophages and T cells, produce TNF-α, which binds to specific receptors on target cells, thereby activating multiple intracellular signaling pathways. Consequently, the production of other pro-inflammatory cytokines, such as IL-1 and IL-6, is induced, along with the recruitment and activation of immune cells, including neutrophils and monocytes [46]. 

TNF-α is recognized as a key contributor to the initiation and persistence of joint inflammation and damage in PsA. It facilitates the production of enzymes that degrade the cartilage and bone, thereby leading to joint destruction. Moreover, TNF-α induces angiogenesis, which further fuels the progression of joint inflammation [2]. Furthermore, TNF-α plays a crucial role in the development of erosions through the induction of receptor activator of nuclear factor-κB (NF-κB) ligand expression in the synovium, a pivotal factor in osteoclast differentiation. Additionally, TNF-α stimulates synovial fibroblasts to express Dickkopf-related protein 1, which inhibits osteoblast differentiation, thereby exacerbating the formation of erosions [47].

### 3.2. Role of IL-23 in PsA

IL-23 is a cytokine composed of two subunits, p19 and p40, with the latter being shared with IL-12. Its main function is to facilitate the activation, differentiation, and proliferation of Th17 cells, which are responsible for producing effector cytokines like IL-17A and IL-22. However, it is worth noting that IL-17 production can also occur independently of IL-23 [48]. The injection of IL-23 causes a skin disease like psoriasis in mice with normal expression of IL-17, while IL-17 knockout mice do not exhibit such disease manifestations. Moreover, the pretreatment of wild-type mice with anti-IL-17A antibodies effectively prevents IL-23-mediated disease development [49]. These findings indicate that IL-23 functions as an upstream regulator of IL-17A, whereas IL-17A, acting downstream, directly impacts tissue physiology.

The physiological processes governed by the IL-23 signaling strongly support its crucial role in maintaining the homeostasis of the intestinal barrier. Intestinal inflammation is linked to the increased permeability of the gut barrier, which facilitates the infiltration of pathogen-associated molecular patterns into the host system. The compromised intestinal barrier leads to a persistent activation of the host’s defense mechanisms, which subsequently triggers an excessive production of IL-23, the cytokine that plays a pivotal role in the development of SpA pathogenesis [50]. 

IL-23 demonstrates a remarkable capacity to induce inflammation in various anatomical sites, including the gut, skin, enthesis, and joints, with high efficiency. Notably, IL-23 alone could directly induce the characteristic clinical features of psoriasis and PsA in mice. Experimental evidence has shown that IL-23 expression in mice results in the emergence of enthesitis, osteitis, new bone formation, and bony erosion [51]. In mice overexpressing IL-23 specifically in the skin, the initial manifestation of psoriatic-like skin lesions was observed, followed by the development of suggestive musculoskeletal features, including enthesitis, synovitis, and dactylitis [52]. IL-23 and its receptor IL-23R appear to play a central role in the pathogenesis of PsA and SpA in general, as they are present in various anatomical sites affected by these conditions [2]. Consequently, various types of immune cells that express IL-23R, including Th17 cells, γ/δ T cells, ILC3s, and CD8^+^IL-23R^+^ mucosal-associated invariant T cells, are elevated in the joints and entheses of SpA and PsA patients [53]. IL-23 plays a crucial role in mediating inflammation in the skin and gut, acting as a significant link between these pathological conditions and peripheral joints. However, its involvement in axial inflammation seems to be relatively minimal [54].

### 3.3. Role of IL-17 in PsA

IL-17, a group of pro-inflammatory cytokines comprising six known members (IL-17 A/B/C/D/E/F), is mainly generated by Th17 T cells, and its presence has been associated with the development of psoriatic skin lesions and enthesitis [55]. IL-17 is secreted by multiple cell populations, including Th17 helper cells, γ/δ T cells, interstitial lymphoid cells, neutrophils, and mast cells [2]. Several studies have suggested that a combination of cytokines, including IL-23, TGF-β, IL-6, IL-1β, and IL-21, can induce the differentiation of naïve T cells into Th17 cells [56]. IL-23 stimulates activated Th17 cells, leading to the release of IL-17. However, clinical trials conducted in human subjects have demonstrated the limited efficacy of IL-23 inhibitors in the treatment of axSpA, suggesting the involvement of other cytokines in IL-17 production. This observation implies that IL-17 can also be generated independently of IL-23 [56].

While many members of the IL-17 family contribute to inflammation and participate in immune-related diseases and defense against pathogens, they also have various functions in preserving mucosal integrity, responding to allergens, supporting anti-inflammatory responses, and regulating the function of lymphocytes [57]. Among these members, IL-17A and IL-17F are particularly significant in PsA. IL-17A and IL-17F have the ability to form both homo- and heterodimers, which can bind to the IL-17 receptor and stimulate signaling pathways downstream. This activation leads to the release of pro-inflammatory cytokines. In PsA, IL-17A is considered the main pro-inflammatory cytokine, although IL-17F likely also has some effects [58]. 

IL-17A has a multitude of effects on diverse immune cells in the skin and joints, stimulating inflammation, coagulation, and causing damage to bones and joints. IL-17A exerts significant biological effects by attracting neutrophils, Th17 cells, and ILC3s to the affected tissues, particularly the skin and entheses. This recruitment process contributes to the generation of the immune response [50]. IL-17A acts as a mediator that aggravates tendon inflammation by initiating neutrophil responses. This inflammatory process clinically presents as enthesitis and tendinitis, which are characteristic features of PsA [59]. Both preclinical and clinical pieces of evidence indicate that bone erosion and new bone formation coexist at various anatomical sites in SpA, and IL-17A appears to have a multifaceted involvement in these mechanisms [60]. IL-17A stimulates stromal cells and macrophages to produce pro-inflammatory cytokines such as TNF, IL-1, and IL-6, which contribute to osteoclastogenesis. Overall, by virtue of its stimulatory effect on osteoclastogenesis, IL-17A plays a catabolic role in bone metabolism. This phenomenon helps elucidate the mechanisms underlying the formation of bone erosions and the occurrence of osteopenia and/or osteoporosis that are commonly observed in patients with PsA [61]. 

### 3.4. Other Cytokines or Chemokines in PsA

While the therapeutic efficacy of apremilast, a phosphodiesterase 4 (PDE4) inhibitor, in PsA is linked to its capacity to suppress the production of TNF-α, IL-17, and IL-23, the effectiveness of Janus kinase (JAK) inhibition in PsA treatment suggests the participation of other cytokines in the inflammatory mechanisms underlying this condition [62,63].

IL-22 is a cytokine generated by Th22 cells, a recently identified subset of T helper cells expressing chemokine receptor (CCR)10. Serum levels of IL-22 are notably elevated in patients with PsA compared to those in patients with psoriasis only [64]. In regulating bone homeostasis, IL-22 has been shown to have dual effects on both osteoclasts and osteoblasts. It has been reported to promote the expression of RANKL and upregulate osteoclastogenesis [65]. On the other hand, IL-22 promotes osteoblast differentiation and function, leading to enhanced bone formation. It can stimulate the production of various osteogenic factors and extracellular matrix proteins by osteoblasts, thereby supporting bone mineralization and overall bone formation [66]. IL-32 activates monocytes, promoting their differentiation into macrophages or DC, and also triggers the production of pro-inflammatory cytokines such as TNF-α, IL-1β, IL-6, and IL-8 through the inflammatory signaling pathway involving NF-κB and p38 mitogen-activated protein kinase [67]. Plasma levels of IL-32 are elevated in patients with PsA compared to those in healthy controls, and notably higher than in patients with psoriasis alone. These findings indicate that plasma IL-32 levels in patients with psoriasis only increase prior to PsA onset, and further elevate upon PsA aggravation [68]. IL-33, a member of the IL-1 family, interacts with the ST2 receptor and activates various immune cells, including ILC2 and Th2 cells. Furthermore, the IL-33/ST2 axis appears to play a role in regulating the Th17 response [69]. Several studies demonstrated the involvement of IL-33 in both psoriasis pathogenesis and PsA progression [70]. Consistently, serum levels of IL-33 in patients with PsA are elevated [71]. However, there is limited evidence suggesting a significant involvement of IL-6, which is a key cytokine in RA, in the development of PsA [72]. 

Prior to treatment, patients with PsA exhibit a notably higher expression of CXCL12 compared to that in patients with psoriasis vulgaris [73]. The significant up-regulation of myeloid cells in PsA has generated increased attention from researchers towards myeloid-derived pro-inflammatory mediators, including osteopontin and CCL2, found in the synovial fluid of patients with PsA [74]. Elevated expression levels of the *CCL1*, *CCL20*, *CXCL5*, and *CX3CL1* genes were detected in both the synovial fluid and peripheral blood cells of individuals with PsA [75]. The levels of CXCL4 in the synovial fluid of patients with PsA were found to correlate significantly with IL-17 and IL-22 levels. Collectively, these findings provided evidence that CXCL4 enhances the production of pro-inflammatory cytokines, particularly IL-17, by human CD4^+^ T cells, suggesting a potential involvement of CXCL4 in the pathology of PsA [76].

## 4. Cytokine-Targeted Therapies in PsA 

In recent years, the development of innovative drugs that specifically inhibit cytokines and intracellular signaling pathways has led to new treatment paradigms for PsA (Table 2). The heterogenous features of PsA pose a challenge for rheumatologists trying to select the optimal treatment for an individual. In order to address these challenges, global organizations including the Group for Research and Assessment of Psoriasis and Psoriatic Arthritis (GRAPPA) and the European League Against Rheumatism (EULAR) have released guidelines to provide guidance for healthcare providers [77,78]. These guidelines prioritize the initial use of conventional synthetic disease-modifying antirheumatic drugs (csDMARDs) with subsequent options including therapy with biological disease-modifying antirheumatic drugs (bDMARDs) or targeted synthetic DMARDs (tsDMARDs), such as JAK inhibitors (JAKi) and PDE4 inhibitors.

For a considerable period, TNF inhibitors (TNFi) have been recognized as the most efficacious treatment for managing the various clinical manifestations of PsA. However, a significant percentage of patients did not achieve a satisfactory response to TNFi. The wide range of novel therapies derived from understanding the IL-23/IL-17 axis in PsA pathogenesis has provided additional treatment choices for PsA patients. However, on a population scale, these therapies have not demonstrated superior overall outcomes compared to the effects of TNFi. As a next therapeutic approach, JAKi may effectively suppress the pro-inflammatory actions of various cytokines and growth factors.

### 4.1. TNF-α Inhibitors in PsA

TNFi, a class of pharmaceuticals that antagonize TNF-α activity, have shown efficacy in the management of PsA. These medications, including etanercept, certolizumab pegol, infliximab, adalimumab, and golimumab, decrease joint inflammation, improve physical function, and prevent joint damage in patients with PsA [79]. TNFi have shown significant efficacy in treating all SpA manifestations and are recommended by the GRAPPA and EULAR for the management of enthesitis and dactylitis [80]. Phase III trials showed TNFi efficacy in PsA with acceptable safety profiles. Significant improvements according to the American College of Rheumatology criteria (ACR20 response) were observed for all TNFi compared to the effects of placebo in PsA. Moreover, all five TNFi inhibited radiographic progression [80].

Among TNFi, etanercept was less effective than TNFi representing monoclonal antibodies in the treatment of uveitis or inflammatory bowel disease, which are extra-musculoskeletal manifestations of PsA [81,82]. On the other hand, etanercept exhibits lower immunogenicity compared to other TNFi and does not necessitate concurrent methotrexate administration to maintain its long-term efficacy. In a randomized, controlled phase III trial, both etanercept monotherapy and combination therapy with etanercept and methotrexate had superior efficacy compared to that achieved with methotrexate monotherapy in patients with PsA. Furthermore, the addition of methotrexate did not synergistically enhance the efficacy of etanercept [83]. 

Nevertheless, there can be adverse effects associated with TNFi, including higher susceptibility to infections, so they necessitate careful supervision by a healthcare professional. The TNFi drugs do not prevent the development of new bone in patients with PsA. In fact, these results suggest that cytokines other than TNF-α may play a crucial role in the abnormal bone growth in PsA [84]. 

### 4.2. IL-17 Inhibitors in Psoriatic Arthritis

The prominent involvement of IL-17 in the immune-related mechanism of PsA validates the use of IL-17 inhibitors for all forms of psoriatic disease. Secukinumab became the first IL-17 inhibitor to receive approval for the treatment of PsA. After that, ixekizumab was approved, and bimekizumab and brodalumab are also being studied for their potential efficacy in PsA.

Secukinumab is a human IgG1k class monoclonal antibody that specifically targets IL-17A. The FUTURE trials have shown its superior efficacy over placebo in multiple domains of PsA, including spondylitis, peripheral arthritis, enthesitis, dactylitis, psoriasis, and nail disease [85,86]. Moreover, secukinumab reduced radiographic progression compared to the effect of placebo. In a head-to-head clinical trial comparing secukinumab and adalimumab, the former did not show superiority in musculoskeletal domain outcomes in patients with PsA. However, therapy with secukinumab was associated with better skin outcomes and a higher treatment retention rate [87].

Ixekizumab is a IgG4κ subclass monoclonal antibody, produced using recombinant technology, which specifically targets IL-17A. In both TNFi refractory and naïve patients, ixekizumab exhibited superiority over the placebo in terms of ACR responses, inhibition of radiographic progression, and various clinical aspects, similar to the effects observed with secukinumab [88,89]. Ixekizumab was superior to adalimumab in achieving the complete remission of psoriasis, as indicated by the psoriasis area and severity index (PASI)100 response at week 24 in a head-to-head clinical trial. However, there were no significant differences in the clinical effect on musculoskeletal domain manifestations between ixekizumab and adalimumab [90].

Bimekizumab, a humanized IgG1κ monoclonal antibody targeting IL-17A and IL-17F, demonstrated notable improvements in the ACR50 response compared to the placebo in PsA patients treated for 12 weeks, as evidenced by data from the randomized controlled trial (BE ACTIVE). Moreover, sustained improvements in pain and fatigue were observed over a period of 3 years [91,92]. In a recent randomized, double-blind, placebo-controlled, phase 3 trial (BE COMPLETE), treatment with bimekizumab resulted in more significant enhancements in joint and skin efficacy measures at week 16 compared to the effects of the placebo in patients with PsA who had previously experienced an inadequate response or intolerance to TNFi [93].

Brodalumab, a human IgG2 antibody, specifically binds to IL-17 receptor A, leading to the inhibition of IL-17A, IL-17F, and IL-17E. In multicenter phase III clinical trials (AMVISION-1 and 2), brodalumab demonstrated a superior ACR response compared to the placebo group at week 24. Additionally, significant improvements in dactylitis, enthesitis, and psoriasis resolution rates were observed in the brodalumab treatment group [94].

IL-17 inhibitors are recommended for the management of most manifestations in PsA. A recent meta-analysis showed similar effectiveness of anti-IL-17A agents and TNFi in the treatment of arthritis, with superior cutaneous responses to anti-IL-17A agents [95]. However, patients with PsA and inflammatory bowel disease (IBD) are an exception, as IL-17 inhibitors in this group are not recommended owing to the lack of demonstrated efficacy and potential exacerbation of IBD manifestations [96]. The production of IL-17 in the gut epithelium occurs independently of IL-23 stimulation and is involved in tissue repair processes and local tissue homeostasis. Therefore, blocking IL-17 may paradoxically trigger or worsen IBD symptoms, making the use of IL-17 inhibitors contraindicated in patients with IBD [97]. Furthermore, in patients with recurrent uveitis, monoclonal antibodies targeting TNF-α are generally preferred over IL-17 inhibitors, as the evidence of the efficacy of the latter treatment is limited [97]. 

### 4.3. IL-23 Inhibitors in PsA

The first IL-23 inhibitor introduced to the market was ustekinumab, which reduces the activities of both IL-23 and IL-12 by specifically targeting their shared p40 subunit. Furthermore, monoclonal antibodies guselkumab, risankizumab, and tildrakizumab specifically target the p19 subunit of IL-23 [98]. 

Ustekinumab effectively inhibits the differentiation of Th1 and Th17 cells. Phase 3 clinical trials (PSUMMIT trials 1 and 2) showed that this drug effectively treated skin manifestations, peripheral arthritis, enthesitis, and dactylitis in both TNFi-experienced and TNFi-naïve patients with PsA [15]. The use of ustekinumab resulted in a higher rate of achieving the primary outcome, defined as an ACR20 response at week 24, compared to the placebo group [99]. Positive responses to the ustekinumab treatment were sustained over a period of 2 years, and there was a significant reduction in radiographic progression in patients with active PsA [100,101]. In the open-label randomized controlled trial ECLIPSA, patients with PsA showed better improvement of enthesitis at week 24 after ustekinumab than after TNFi [102]. Furthermore, the results of an observational prospective real-world study indicated that TNFi and ustekinumab yielded comparable responses in terms of achieving low disease activity or minimal disease activity after 6 months. Additionally, the study revealed similar safety outcomes for both treatments [103]. In the management of psoriasis, ustekinumab was more effective than etanercept, a soluble TNF receptor inhibitor. Nevertheless, subsequent studies have suggested that targeting IL-23 through p19 inhibition had superior effectiveness in comparison to the effects of p40 inhibition for psoriasis treatment. These findings imply that IL-12 may play a protective role in psoriasis. Due to the favorable outcomes observed with p19 inhibition in psoriasis, it has become the preferred approach for inhibiting IL-23 in dermatology practice. This shift in preference is a result of the significant efficacy of p19 inhibition in treating psoriasis [98,104].

Guselkumab, a monoclonal antibody that specifically binds to the p19 subunit and blocks the activity of IL-23, has obtained approval for the treatment of PsA. In clinical trials (DISCOVER 1&2), the ACR20 response to guselkumab was significantly higher than to the placebo at week 24 both in TNFi-naïve and TNFi-treated PsA patients [105,106,107]. The safety and clinical improvements were consistently maintained over 2 years [108]. Guselkumab rapidly decreased the levels of type 17 effector cytokines as early as week 4 after the start of the treatment. After 24 weeks of the treatment with guselkumab, the cytokine levels reached levels similar to those found in healthy individuals without PsA, indicating normalization of the IL-23/IL-17 effector cytokines. Notably, the decreases in IL-17A and IL-17F levels were more pronounced in patients treated with guselkumab than in those who received ustekinumab [109]. 

Risankizumab, a monoclonal antibody targeting IL-23, has received the U.S Food and Drug Administration (FDA) approval for the treatment of PsA. The clinical trials conducted on both bDMARD-naïve and bDMARD-experienced patients with PsA showed that significantly more patients achieved an ACR20 response at week 24 after the treatment with risankizumab compared to the effects of the placebo [110,111]. Tildrakizumab, another monoclonal antibody targeting IL-23, has shown promising efficacy against PsA in a phase 2 trial. However, although patients in the tildrakizumab groups demonstrated higher response rates in skin-related outcomes, there were no positive outcomes for enthesitis and dactylitis [112].

Neither of the IL-23 inhibitors has met the primary endpoint to be a considered as a treatment option for axSpA [15]. However, a post hoc analysis indicated the potential efficacy of guselkumab in treating axial involvement in PsA [113]. It should be noted that IL-23 inhibitors generally have a good safety profile, but further evidence from long-term extension studies is needed to fully assess their benefits and risks.

### 4.4. JAKi in PsA

The Janus kinase-signal transducer and activator of transcription (JAK-STAT) pathway plays a crucial role in the activation of the immune system in response to cytokines and growth factors, facilitating the transmission of signals from cell-membrane receptors to the nucleus. This pathway involves four JAK proteins, namely JAK1, JAK2, JAK3, and tyrosine-protein kinase 2 (TYK2). These molecules intricately interact with different members of the signal transducers and activators of the transcription (STAT) family, ultimately regulating the transcription of genes downstream of the pathway [114]. These signaling pathways promote the proliferation and activation of T helper cells and stimulate the expression of IL-17 and IL-23 receptors in the skin and joints. This emphasizes their significance in the development of PsA.

Tofacitinib is a small molecule that selectively blocks the activities of JAK1 and JAK3. In two separate phase III trials with a randomized, double-blind design for PsA patients, ACR20 response rates and improvement in the health assessment questionnaire–disability index scores were significantly greater after tofacitinib than after the placebo [115,116]. The tofacitinib group showed improved clinical outcomes of peripheral arthritis, dactylitis, enthesitis, and psoriasis compared to the outcomes in the placebo group during the first 6 months of therapy [117]. Furthermore, in the long-term extension study, tofacitinib attenuated radiographic progression in patients with axial involvement [117]. In terms of safety, the ORAL Surveillance Study sparked controversy by suggesting that the use of tofacitinib in patients with RA may increase the risk of cancer and cardiovascular diseases [118]. Consequently, both the European Medicines Agency (EMA) and the FDA have warned against the initial use of JAKi in patients with a high risk of cardiovascular event or malignancy.

Upadacitinib, a highly specific inhibitor of JAK1, has recently been approved as the latest drug in its class for the treatment of PsA. The phase 3 clinical trial SELECT-PsA 1 provided evidence of the effectiveness of upadacitinib in achieving a significant ACR20 response when compared to the effects of placebo. Significant improvements in secondary endpoints, such as decreased radiographic progression, attainment of minimal disease activity, resolution of dactylitis, and improvement in enthesitis, were observed in both the 15 and 30 mg upadacitinib treatment groups, surpassing the results seen in the placebo group. Furthermore, upadacitinib at doses of 15 and 30 mg had comparable or higher efficacy than adalimumab. However, the 30 mg dose of upadacitinib was associated with a higher incidence of severe adverse events. Additionally, both the upadacitinib and adalimumab treatment groups had comparable PsA manifestations, such as psoriasis, enthesitis, physical function deficit, fatigue, and lower quality of life [119,120,121]. Similarly, patients who were non-responsive or intolerant to TNFi (SELECT-PsA2) had significantly greater ACR20 responses to upadacitinib and achieved minimal disease activity compared to that noted in the placebo group [122].

Filgotinib, another selective JAK1 inhibitor, had notable superiority over the placebo in the randomized, double-blind, phase 2 trial EQUATOR for PsA. That study revealed that a considerably higher proportion of patients who received filgotinib achieved the primary endpoint of ACR20 response at week 16. Additionally, filgotinib administration led to superior improvements in peripheral arthritis, enthesitis, psoriasis, physical functioning, fatigue, and pain [123]. However, preclinical studies have raised concerns regarding potential adverse effects on male patients’ reproductive function [124]. Some results from the safety studies will be needed to relieve this concern. 

Deucravacitinib is a small molecule that selectively and non-competitively inhibits TYK2. By binding to the regulatory domain of TYK2, it modulates the immune inflammatory response to IL-12, IL-23, and type I IFNs [125]. In a phase II clinical trial involving patients with active PsA, significant improvements in arthritis, enthesitis, dactylitis, and psoriasis domains were observed in participants receiving deucravacitinib at two different doses (6 mg and 12 mg QD) compared to the effects in patients receiving placebo. A post hoc analysis conducted on data from a phase 2 trial of deucravacitinib examined the ACR20 response at week 16 in patients with PsA, considering their prior exposure to csDMARDs. These findings revealed that all patients treated with deucravacitinib, regardless of their prior exposure to csDMARDs, demonstrated comparable clinical improvements [126]. Currently, a phase 3 randomized controlled trial of deucravacitinib for PsA is ongoing.

Brepocitinib is an orally administered drug with dual inhibitory activity toward TYK2 and JAK1. New findings from a phase 2 randomized controlled trial indicated that both 30 mg and 60 mg doses of brepocitinib administered once daily were more effective in alleviating PsA signs and symptoms than the placebo. Over the course of a 52-week study, brepocitinib had a favorable safety profile and was well-tolerated by the participants [127].

Recent advancements in our understanding of the JAK/STAT pathway have enabled the development of two JAK inhibitors (JAKi) that have received approval for the treatment of PsA: tofacitinib and upadacitinib. These JAKi showed both a favorable safety profile and efficacy, leading to rapid improvements in PsA symptoms, quality of life, and attenuation of radiographic progression. Notably, when compared to other b/tsDMARDs, JAKi offer several advantages, namely a short half-life, oral route of administration, and better patient compliance [125]. TYK2 inhibitors, in particular, have shown a favorable safety profile, possibly attributed to their highly selective mode of action [126]. It will be of the utmost importance in the future to study these treatments in a real-world setting to address the unmet requirements of all individuals with PsA and provide answers to questions that clinical trial data alone may not fully address.

### 4.5. Other Pharmacological Treatments

Apremilast, an orally administered small molecule that functions as a PDE4 inhibitor, obtained FDA approval in 2014 for the management of PsA. The inhibition of PDE4 elevates intracellular cyclic AMP levels, which subsequently reduces the synthesis of pro-inflammatory cytokines and enhances the production of anti-inflammatory cytokines such as IL-10 [15]. The efficacy of apremilast in PsA has been shown in the PALACE trials [62,128,129,130]. The GRAPPA guidelines suggest the use of apremilast for patients presenting with psoriasis, nail involvement, peripheral arthritis, enthesitis, and dactylitis.

Although numerous drugs may effectively treat PsA, some patients fail to respond, lose response over time, or experience drug toxicity, resulting in discontinuation of the medication or the need for combination therapies. Consequently, there remains a significant demand for new alternative treatments [131]. 

Remtolumab is a dual variable domain immunoglobulin that binds to both TNF-α and IL-17A. It was expected that a drug targeting two key cytokines simultaneously would be able to treat PsA more effectively. Nonetheless, the ACR20 and PASI90 responses to remtolumab and adalimumab were comparable. As a result, the approach of simultaneously inhibiting both the TNF-α and IL-17A pathways was abandoned, even though treatment with dual variable domain immunoglobulin appeared promising for patients with challenging-to-treat PsA [132].

Nanobodies, which are antibodies consisting of the recombinant variable domain on a heavy chain, could be another promising tool for new drug development. Nanobodies possess several useful characteristics such as small size, good solubility, high stability, easy production, fast elimination from the bloodstream, and effective penetration into deep tissues. These properties make nanobodies highly valued as therapeutic tools, and they are also being explored for use in chimeric antigen receptors and targeted drug delivery systems [133]. A phase 3 clinical trial for netakimab, a recombinant IgG1 nanobody targeting IL-17A, is ongoing in patients with PsA [134]. Sonelokimab is an innovative trivalent nanobody that consists of three distinct parts. The C-terminal segment of this drug attaches to both IL-17A and IL-17F subtypes, while the middle segment binds to serum albumin. The N-terminal segment, on the other hand, selectively targets IL-17F [135]. Although the effectiveness and safety of these two drugs have been demonstrated in patients with plaque psoriasis, there are currently no published data evaluating their effects specifically on patients with PsA.

## 5. Future Perspectives and Conclusions

The progress in the elucidation of the impacts of inflammatory cytokines on PsA pathogenesis has enabled exploration of the novel molecular mechanisms that can be precisely targeted by therapeutic approaches. The activation of immunoinflammatory pathways in PsA involves multiple cell types and pro-inflammatory cytokines, such as TNF-α, IL-17, and IL-23. Providing timely and effective treatment is of the utmost importance in preventing the spread of joint damage and improving the overall well-being of individuals diagnosed with PsA. Despite the advancements in understanding PsA pathogenesis and the development of new drugs that target specific cytokines, managing PsA continues to pose challenges for rheumatologists.

**Figure 1:**
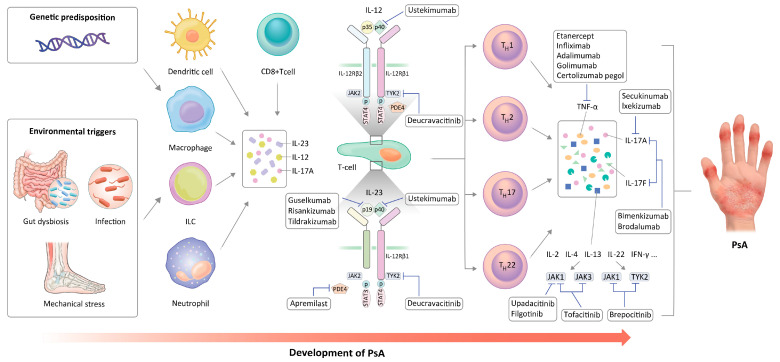
Pathogenesis of PsA and therapeutic targets.

The pathogenesis and current therapeutic targets of PsA are shown in Figure 1. Our review shows that the ongoing studies investigating PsA pathogenesis offer the potential to reveal more pertinent therapeutic targets and broaden the array of available treatment options. Furthermore, there is a burgeoning interest in utilizing new biotechnology tools and combination therapies aimed at targeting multiple pathogenic pathways to achieve more effective disease control. Although there is little reliable clinical evidence of new drugs surpassing previous b/tsDMARDs in efficacy, significant advancements achieved in recent years in this field offer a promising outlook for the future.

## Figures and Tables

**Table 1:** Inflammatory cytokines in PsA.

Cytokine	Main Source	Function in PsA
TNF-α	Macrophages, T cells, NK cells, mast cells	Elevation in the levels of cytokines, chemokines, matrix metalloproteinases, and adhesion molecules from various immune cells Induction of osteoclasts to promote the degradation of cartilage and bone
IL-23	Dendritic cells, macrophages	Promotion of Th17 cell differentiation and GM-CSF production
IL-12	Dendritic cells, macrophages	Promotion of Th1 cell differentiation through STAT4
IL-17A/F	Th17 cells, NK cells, type 3 innate lymphoid cells	Stimulation of fibroblast-like synoviocytes, chondrocytes, and osteoclasts Elevation in the levels of pro-inflammatory cytokines and matrix metalloproteinases Induction of neutrophil recruitment
IL-21	T cells, NK cells	Promotion of T cell differentiation (specifically, into Th17 cells)
IL-22	T cells, innate lymphoid cells	Activation of fibroblast-like synoviocytes Promotion of osteoclast and bone degradation
IL-32	NK cells, T cells, monocytes, epithelial cells	Potentiation of inflammation through the activation of NF-κB signaling and promotion of osteoclast differentiation
IL-33	Macrophages, dendritic cells, mast cells, epithelial cells	Activation of Th1/Th17-mediated inflammation
IFN γ	Th1 cells, type 1 innate lymphoid cells, NK cells	Activation of macrophage and T cell Promotion of RANKL secretion
GM-CSF	Macrophages, T cells, synovial fibroblasts	Recruitment of various immune cells
IL-9	Th9 cells	Promotion of Th17-associated inflammation and IL-17A production
IL-6	Macrophages, T cells, endothelial cells	Stimulation of STAT3 signaling to increase the production of pro-inflammatory cytokines
IL-15	Macrophages	Promotion of T cell proliferation; natural killer cell activation; and production of IFN-γ, TNF-α, and IL-17
IL-1α	Macrophages, dendritic cells	Induction of IL-17, IL-21, and IL-22 expression by γδT cells in combination with IL-23

GM-CSF: granulocyte-macrophage colony-stimulating factor; IFN: interferon; IL: interleukin; RANKL: receptor activator of nuclear factors’ κB ligand; STAT: signal transducer and activator of transcription; TNF: tumor necrosis factor.

**Table 2:** Current cytokine-targeting treatment for psoriatic arthritis.

b/tsDMARDs	Target	Dose and Administration Route	FDA or EMA Approval
Etanercept	TNF-α	50 mg weekly, SC	FDA, EMA
Infliximab	TNF-α	5 mg/kg at weeks 0, 2, and 6; every 8 weeks thereafter, IV	FDA, EMA
Adalimumab	TNF-α	40 mg every 2 weeks, SC	FDA, EMA
Golimumab	TNF-α	50 mg weekly, SC	FDA, EMA
Certolizumab pegol	TNF-α	200 mg every 2 weeks or 400 mg every 4 weeks, SC	FDA, EMA
Ustekinumab	p40 subunit of IL-12 and IL-23	(BW < 100 kg) 45 mg/kg at weeks 0, 4, and 12; every 12 weeks thereafter, SC (BW > 100 kg) 90 mg/kg at weeks 0, 4, and 12; every 12 weeks thereafter, SC	FDA, EMA
Guselkumab	p19 subunit of IL-23	100 mg at weeks 0 and 4; every 8 weeks thereafter, SC	FDA, EMA
Risankizumab	p19 subunit of IL-23	150 mg at weeks 0 and 4; every 12 weeks thereafter, SC	FDA, EMA
Tildrakizumab	p19 subunit of IL-23	100 mg at weeks 0 and 4; every 12 weeks thereafter, SC	-
Secukinumab	IL-17A	150 mg weekly for 4 weeks; monthly thereafter, SC	FDA, EMA
Ixekizumab	IL-17A	160 mg at weeks 0 and 80 mg at weeks 2, 4, 6, 8, 10, and 12; 80 mg every 4 weeks thereafter, SC	FDA, EMA
Bimekizumab	IL-17A/F	320 mg every 4 weeks for 16 weeks, every 8 weeks thereafter, SC	EMA
Brodalumab	IL-17 receptor	210 mg at weeks 0, 1, and 2; every 2 weeks thereafter, SC	-
Tofacitinib	JAK1/3	5 mg twice daily, orally	FDA, EMA
Upadacitinib	JAK1	15 mg once daily, orally	FDA, EMA
Filgotinib	JAK1	200 mg once daily, orally	-
Deucravacitinib	TYK2	6 mg once daily, orally	-
Brepocitinib	JAK1/TYK2	30 mg or 60 mg once daily, orally	-

b/tsDMARDs, biological/targeted synthetic disease-modifying anti-rheumatic drugs; EMA, the European Medicines Agency; FDA, the U.S Food and Drug Administration; IV, intravenously; SC, subcutaneously.

## Data Availability

No new data were created or analyzed in this study. Data sharing is not applicable to this article.
